# Supplementary material for: Bio-Computational Evaluation of Compounds of Bacopa Monnieri as a Potential Treatment for Schizophrenia
Source: Molecules. 2022 Oct 19;27(20):7050. doi: 10.3390/molecules27207050 (PMC9611144; doi:10.3390/molecules27207050)
Supplement: Supplementary file 1 [file molecules-27-07050-s001.zip › Figure S1.pdf]

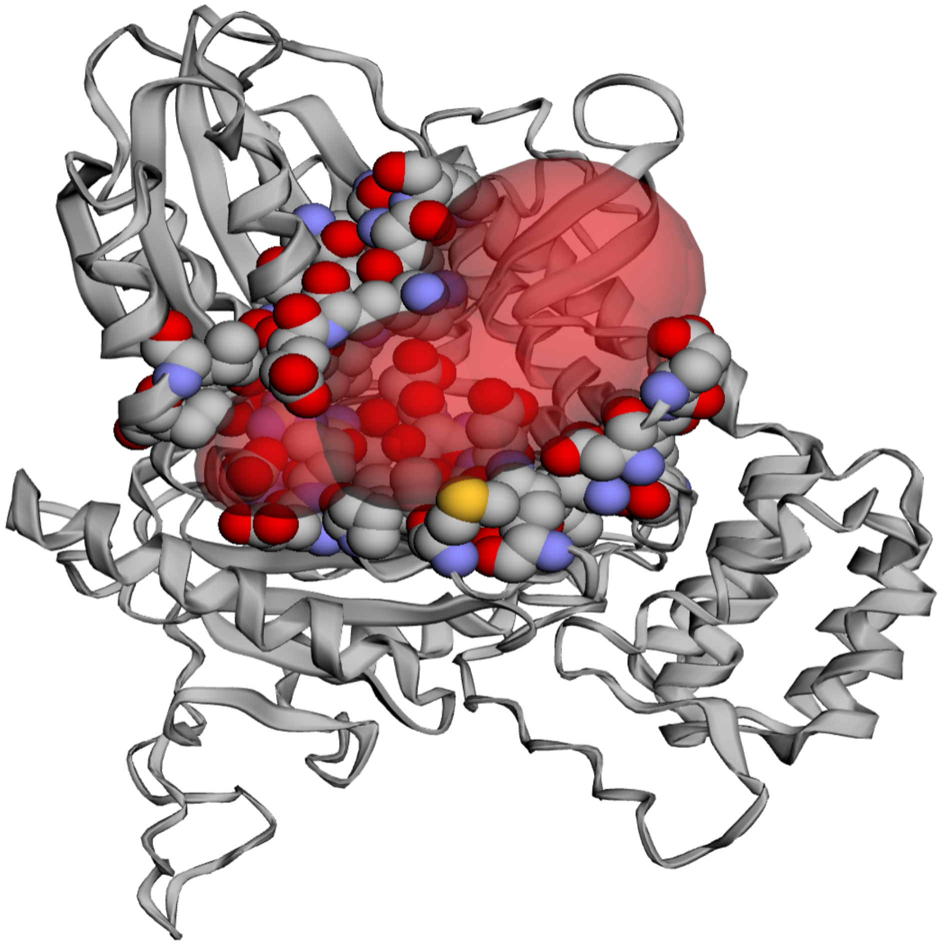

Figure S1: Depicting the 3D visualization of predicted active site (in red sphere) key residues are shown by sphere pattern. STXBP1 shown by grey color in ribbon pattern.
