# Supplementary material for: Bio-Computational Evaluation of Compounds of Bacopa Monnieri as a Potential Treatment for Schizophrenia
Source: Molecules. 2022 Oct 19;27(20):7050. doi: 10.3390/molecules27207050 (PMC9611144; doi:10.3390/molecules27207050)
Supplement: Supplementary file 1 [file molecules-27-07050-s001.zip › Supplementary file S1.pdf]

BIOZENTRUM University of Basel The Center for Molecular Life Sciences

SWISS-MODEL Homology Modelling Report

Model Building Report

This document lists the results for the homology modelling project "P61764 · STXBP1_HUMAN" submitted to SWISS-MODEL workspace on July 1, 2022, 1:30 p.m..The submitted primary amino acid sequence is given in Table T1.

If you use any results in your research, please cite the relevant publications:

- Waterhouse, A., Bertoni, M., Bienert, S., Studer, G., Tauriello, G., Gumienny, R., Heer, F.T., de Beer, T.A.P., Rempfer, C., Bordoli, L., Lepore, R., Schwede, T. SWISS-MODEL: homology modelling of protein structures and complexes. Nucleic Acids Res. 46(W1), W296-W303 (2018). doi>
- Bienert, S., Waterhouse, A., de Beer, T.A.P., Tauriello, G., Studer, G., Bordoli, L., Schwede, T. The SWISS-MODEL Repository - new features and functionality. Nucleic Acids Res. 45, D313-D319 (2017). doi>
- Studer, G., Tauriello, G., Bienert, S., Biasini, M., Johner, N., Schwede, T. ProMod3 - A versatile homology modelling toolbox. PLOS Comp. Biol. 17(1), e1008667 (2021). doi>
- Studer, G., Rempfer, C., Waterhouse, A.M., Gumienny, G., Haas, J., Schwede, T. QMEANDisCo - distance constraints applied on model quality estimation. Bioinformatics 36, 1765-1771 (2020). doi>
- Bertoni, M., Kiefer, F., Biasini, M., Bordoli, L., Schwede, T. Modeling protein quaternary structure of homo- and hetero-oligomers beyond binary interactions by homology. Scientific Reports 7 (2017). doi>

Results

The SWISS-MODEL template library (SMTL version 2022-06-29, PDB release 2022-06-24) was searched with BLAST (Camacho et al.) and HHblits (Steinegger et al.) for evolutionary related structures matching the target sequence in Table T1. For details on the template search, see Materials and Methods. Overall 50 templates were found (Table T2).

Models

The following model was built (see Materials and Methods "Model Building"):

Model #01	File	Built with	Oligo-State	Ligands	GMQE	QMEANDisCo Global
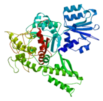	PDB	ProMod3 3.2.1	monomer	None	0.86	0.82 ± 0.05

Template	Seq Identity	Oligo-state	QSQE	Found by	Method	Resolution	Seq Similarity	Range	Coverage	Description
4jeh.1.A	100.00	monomer	0.00	HHblits	X-ray	2.50Å	0.61	4 - 592	1.00	Syntaxin-binding protein 1

The template contained no ligands.

```
Target    MAPIGLKAVVGEKIMHDVIKKVKKKGEWKVLVVDQLSMRMLSSCCKMTDIMTEGITIVEDINKRREPLPSLEAVYLITPS
4jeh.1.A  MAPIGLKAVVGEKIMHDVIKKVKKKGEWKVLVVDQLSMRMLSSCCKMTDIMTEGITIVEDINKRREPLPSLEAVYLITPS

Target    EKSVHSLISDFKDPPTAKYRAAHVFFTDSCPDALFNELVKSRAAKVIKTLTEINIAFLPYESQVYSLDSADSFQSFYSPH
4jeh.1.A  EKSVHSLISDFKDPPTAKYRAAHVFFTDSCPDALFNELVKSRAAKVIKTLTEINIAFLPYESQVYSLDSADSFQSFYSPH

Target    KAQMKNPILERLAEQIATLCATLKEYPAVRYRGEYKDNALLAQLIQDKLDAYKADDPTMGEGPDKARSQLLILDRGFDPS
4jeh.1.A  KAQMKNPILERLAEQIATLCATLKEYPAVRYRGEYKDNALLAQLIQDKLDAYKADDPTMGEGPDKARSQLLILDRGFDPS

Target    SPVLHELTFQAMSYDLLPIENDVYKYETSGIGEARVKEVLLDEDDDLWIALRHKHIAEVSQEVTRSLKDFSSSKRMNTGE
4jeh.1.A  SPVLHELTFQAMSYDLLPIENDVYKYETSGIGEARVKEVLLDEDDDLWIALRHKHIAEVSQEVTRSLKDFSSSKRMNTGE

Target    KTTMRDLSQMLKKMPQYQKELSKYSTHLHLAEDCMKHYQGTVDKLCRVEQDLAMGTDAEGEKIKDPMRAIVPILLDANVS
4jeh.1.A  KTTMRDLSQMLKKMPQYQKELSKYSTHLHLAEDCMKHYQGTVDKLCRVEQDLAMGTDAEGEKIKDPMRAIVPILLDANVS
```

```
Target     TYDKIRIILLYIFLKNGITEENLNKLIQHAQIPPEDSEIITNMAHLGVPIVTDSTLRRRSKPERKERISEQTYQLSRWTP
4jeh.1.A   TYDKIRIILLYIFLKNGITEENLNKLIQHAQIPPEDSEIITNMAHLGVPIVTDSTLRRRSKPERKERISEQTYQLSRWTP

Target     IIKDIMEDTIEDKLDTKHYPYISTRSSASFSTTAVSARYGHWHKNKAPGEYRSGPRLIIFILGGVSLNEMRCAYEVTQAN
4jeh.1.A   IIKDIMEDTIEDKLDTKHYPYISTRSSASFSTTAVSARYGHWHKNKAPGEYRSGPRLIIFILGGVSLNEMRCAYEVTQAN

Target     GKWEVLIGSTHILTPQKLLDTLKKLNKTDEEISS
4jeh.1.A   GKWEVLIGSTHILTPQKLLDTLKKLNKTDEEISS
```

Materials and Methods

Template Search

Template search with BLAST and HHblits has been performed against the SWISS-MODEL template library (SMTL, last update: 2022-06-29, last included PDB release: 2022-06-24).

The target sequence was searched with BLAST against the primary amino acid sequence contained in the SMTL. A total of 26 templates were found.

An initial HHblits profile has been built using the procedure outlined in (Steinegger et al.), followed by 1 iteration of HHblits against Uniclust30 (Mirdita, von den Driesch et al.). The obtained profile has then be searched against all profiles of the SMTL. A total of 33 templates were found.

Template Selection

For each identified template, the template's quality has been predicted from features of the target-template alignment. The templates with the highest quality have then been selected for model building.

Model Building

Models are built based on the target-template alignment using ProMod3 (Studer et al.). Coordinates which are conserved between the target and the template are copied from the template to the model. Insertions and deletions are remodelled using a fragment library. Side chains are then rebuilt. Finally, the geometry of the resulting model is regularized by using a force field.

Model Quality Estimation

The global and per-residue model quality has been assessed using the QMEAN scoring function (Studer et al.).

Ligand Modelling

Ligands present in the template structure are transferred by homology to the model when the following criteria are met: (a) The ligands are annotated as biologically relevant in the template library, (b) the ligand is in contact with the model, (c) the ligand is not clashing with the protein, (d) the residues in contact with the ligand are conserved between the target and the template. If any of these four criteria is not satisfied, a certain ligand will not be included in the model. The model summary includes information on why and which ligand has not been included.

Oligomeric State Conservation

The quaternary structure annotation of the template is used to model the target sequence in its oligomeric form. The method (Bertoni et al.) is based on a supervised machine learning algorithm, Support Vector Machines (SVM), which combines interface conservation, structural clustering, and other template features to provide a quaternary structure quality estimate (QSQE). The QSQE score is a number between 0 and 1, reflecting the expected accuracy of the interchain contacts for a model built based a given alignment and template. Higher numbers indicate higher reliability. This complements the GMQE score which estimates the accuracy of the tertiary structure of the resulting model.

References

- **BLAST**
  Camacho, C., Coulouris, G., Avagyan, V., Ma, N., Papadopoulos, J., Bealer, K., Madden, T.L. BLAST+: architecture and applications. BMC Bioinformatics 10, 421-430 (2009). doi>

- **HHblits**
  Steinegger, M., Meier, M., Mirdita, M., Vöhringer, H., Haunsberger, S. J., Söding, J. HH-suite3 for fast remote homology detection and deep protein annotation. BMC Bioinformatics 20, 473 (2019).
- **Uniclust30**
  Mirdita, M., von den Driesch, L., Galiez, C., Martin, M.J., Söding, J., Steinegger, M. Uniclust databases of clustered and deeply annotated protein sequences and alignments. Nucleic Acids Research 45, D170–D176 (2016).

Table T1:

Primary amino acid sequence for which templates were searched and models were built.

```
MAPIGLKAVVGEKIMHDVIKKVKKKGEWKVLVVDQLSMRMLSSCCKMTDIMTEGITIVEDINKRREPLPSLEAVYLITPSEKSVHSLISDFKDPPTAKYR
AAHVFFTDSCPDALFNELVKSRAAKVIKTLTEINIAFLPYESQVYSLDSADSFQSFYSPHKAQMKNPILERLAEQIATLCATLKEYPAVRYRGEYKDNAL
LAQLIQDKLDAYKADDPTMGEGPDKARSQLLILDRGFDPSSPVLHELTFQAMSYDLLPIENDVYKYETSGIGEARVKEVLLDEDDDLWIALRHKHIAEVS
QEVTRSLKDFSSSKRMNTGEKTTMRDLSQMLKKMPQYQKELSKYSTHLHLAEDCMKHYQGTVDKLCRVEQDLAMGTDAEGEKIKDPMRAIVPILLDANVS
TYDKIRIILLYIFLKNGITEENLNKLIQHAQIPPEDSEIITNMAHLGVPIVTDSTLRRRSKPERKERISEQTYQLSRWTPIIKDIMEDTIEDKLDTKHYP
YISTRSSASFSTTAVSARYGHWHKNKAPGEYRSGPRLIIFILGGVSLNEMRCAYEVTQANGKWEVLIGSTHILTPQKLLDTLKKLNKTDEEISS
```

Table T2:

Template	Seq Identity	Oligo-state	QSQE	Found by	Method	Resolution	Seq Similarity	Coverage	Description
4jeh.1.A	100.00	monomer	-	HHblits	X-ray	2.50Å	0.61	1.00	Syntaxin-binding protein 1
3c98.1.A	100.00	monomer	-	HHblits	X-ray	2.60Å	0.61	1.00	Syntaxin-binding protein 1
7udb.1.A	99.83	monomer	-	HHblits	EM	NA	0.61	1.00	Syntaxin-binding protein 1
7udc.1.A	99.83	monomer	-	HHblits	EM	NA	0.61	1.00	Syntaxin-binding protein 1
3puj.1.A	100.00	monomer	-	HHblits	X-ray	3.31Å	0.61	1.00	Syntaxin-binding protein 1
3puj.2.A	100.00	monomer	-	HHblits	X-ray	3.31Å	0.61	1.00	Syntaxin-binding protein 1
6lpc.1.A	99.66	homo-dimer	0.66	HHblits	X-ray	3.40Å	0.61	1.00	Syntaxin-binding protein 1
6lpc.1.B	99.66	homo-dimer	0.62	HHblits	X-ray	3.40Å	0.61	1.00	Syntaxin-binding protein 1

The table above shows the top 8 filtered templates. A further 25 templates were found which were considered to be less suitable for modelling than the filtered list.
1epu.1.A, 1fvf.1.A, 1fvf.1.B, 1fvh.1.A, 1mqs.1.A, 1tqj.1.A, 1y9j.1.A, 2xhe.1.A, 3f4w.1.A, 3puk.1.A, 3puk.2.A, 4bx8.1.A, 4bx9.1.A, 4bx9.2.A, 4cca.1.A, 4jc8.2.A, 4jc8.3.A, 4jeu.1.A, 4kmo.1.A, 5buz.1.A, 5bv0.1.A, 5bv1.2.A, 5umf.1.A, 6xjl.1.A, 6xm1.1.A
