# Supplementary material for: Bio-Computational Evaluation of Compounds of Bacopa Monnieri as a Potential Treatment for Schizophrenia
Source: Molecules. 2022 Oct 19;27(20):7050. doi: 10.3390/molecules27207050 (PMC9611144; doi:10.3390/molecules27207050)
Supplement: Supplementary file 1 [file molecules-27-07050-s001.zip › Table S1.pdf]

Table S1: List of Neurorelase Cycle Genes Extracted from Reactome Database

**MoleculeType**	**Identifier**	**MoleculeName**
Proteins	P61764-1	UniProt:P61764-1 STXBP1
Proteins	O14810	UniProt:O14810 CPLX1
Proteins	Q86UR5	UniProt:Q86UR5 RIMS1
Proteins	P60880	UniProt:P60880 SNAP25
Proteins	Q9H598	UniProt:Q9H598 SLC32A1
Proteins	P20336	UniProt:P20336 RAB3A
Proteins	P21579	UniProt:P21579 SYT1
Proteins	Q05329	UniProt:Q05329 GAD2
Proteins	Q99259	UniProt:Q99259 GAD1
Proteins	P63027	UniProt:P63027 VAMP2
Proteins	P11142	UniProt:P11142 HSPA8
Proteins	Q9H3Z4	UniProt:Q9H3Z4 DNAJC5
Proteins	Q16623	UniProt:Q16623 STX1A
Proteins	P51649	UniProt:P51649 ALDH5A1
Proteins	P80404	UniProt:P80404 ABAT
Proteins	Q9NSD5	UniProt:Q9NSD5 SLC6A13
Proteins	P48066	UniProt:P48066 SLC6A11
Proteins	P30531	UniProt:P30531 SLC6A1
Proteins	P48065	UniProt:P48065 SLC6A12
Proteins	O15245	UniProt:O15245 SLC22A1
Proteins	O15244	UniProt:O15244 SLC22A2
Proteins	O95153	UniProt:O95153 TSPOAP1
Proteins	O14795	UniProt:O14795 UNC13B
Proteins	O75334	UniProt:O75334 PPFIA2
Proteins	O75335	UniProt:O75335 PPFIA4
Proteins	O75145	UniProt:O75145 PPFIA3
Proteins	Q13136	UniProt:Q13136 PPFIA1
Proteins	Q05940	UniProt:Q05940 SLC18A2
Proteins	P21397	UniProt:P21397 MAOA
Proteins	Q16572	UniProt:Q16572 SLC18A3
Proteins	P28329	UniProt:P28329 CHAT
Proteins	Q9GZV3	UniProt:Q9GZV3 SLC5A7
Proteins	P17600	UniProt:P17600 SYN1
Proteins	Q92777	UniProt:Q92777 SYN2
Proteins	O14994	UniProt:O14994 SYN3
Proteins	O94925	UniProt:O94925 GLS
Proteins	Q9UI32	UniProt:Q9UI32 GLS2
Proteins	Q96QD8	UniProt:Q96QD8 SLC38A2
Proteins	P48664	UniProt:P48664 SLC1A6
Proteins	P43004	UniProt:P43004 SLC1A2
Proteins	P43003	UniProt:P43003 SLC1A3
Proteins	P43005	UniProt:P43005 SLC1A1
Proteins	O00341	UniProt:O00341 SLC1A7
Proteins	O75915	UniProt:O75915 ARL6IP5
Proteins	Q9P2U7	UniProt:Q9P2U7 SLC17A7
Proteins	Q02410	UniProt:Q02410 APBA1
Proteins	O14936	UniProt:O14936 CASK
Proteins	O14910	UniProt:O14910 LIN7A
Proteins	Q9HAP6	UniProt:Q9HAP6 LIN7B
Proteins	Q9NUP9	UniProt:Q9NUP9 LIN7C

Proteins	Q02083	UniProt:Q02083 NAAA
