# Supplementary material for: Bio-Computational Evaluation of Compounds of Bacopa Monnieri as a Potential Treatment for Schizophrenia
Source: Molecules. 2022 Oct 19;27(20):7050. doi: 10.3390/molecules27207050 (PMC9611144; doi:10.3390/molecules27207050)
Supplement: Supplementary file 1 [file molecules-27-07050-s001.zip › Table S2.pdf]

Table S2: physicochemical qualities assessment of Bacopa natural compounded. Data obtained

Molecule Name	UNIQUE_SMILES	USER_SUPPLIED_SMILES
C7H8O2	OCc1ccc(O)cc1	C1=CC(=CC=C1CO)O
C7H6O3	[OH2+]c1ccc(cc1)C([O-])=	C1=CC(=CC=C1C(=O)[O-])[OH2+]
C11H16O3	CC1(C)C[C@H](O)C[C@@	C[C@@]12C[C@H](CC(C1=CC(=O)O2)(C)
C11H12O4	COc1ccc(C=CC(O)=O)cc1	COC1=C(C=C(C=C1)C=CC(=O)O)OC
C11H12O4	COc1ccc(\C=C\C(O)=O)cc	COC1=C(C=C(C=C1)/C=C/C(=O)O)OC
C15H10O5	Oc1ccc(cc1)C2=CC(=O)c3	C1=CC(=CC=C1C2=CC(=O)C3=C(C=C(C=C
C15H10O6	Oc1cc(O)c2C(=O)C=C(Oc2	C1=CC(=C(C=C1C2=CC(=O)C3=C(C=C(C=
C14H26O6	CCCCC[C@@H](O[C@@H	CCCCC[C@H](C=C)O[C@H]1[C@@H]([C
C14H26O6	CCCCCC(OC1OC(CO)C(O)	CCCCCC(C=C)OC1C(C(C(C(O1)CO)O)O)O
C15H10O7	Oc1cc(O)c2C(=O)C(=C(Oc	C1=CC(=C(C=C1C2=C(C(=O)C3=C(C=C(C=
C14H20O8	OC[C@H]1O[C@@H](OC	C1=CC(=C(C=C1CCO[C@H]2[C@@H]([C
C14H20O8	OCC1OC(OCCc2ccc(O)c(O	C1=CC(=C(C=C1CCOC2C(C(C(C(O2)CO)O)
C16H20O9	COc1cc(\C=C\C(=O)O[C@	COC1=C(C=CC(=C1)/C=C/C(=O)O[C@H]2
C14H26O9S	CCCCC[C@@H](O[C@@H	CCCCC[C@H](C=C)O[C@H]1[C@@H]([C
C14H26O9S	CCCCCC(O[C@@H]1O[C@	CCCCCC(C=C)O[C@H]1[C@@H]([C@H](
C28H46O	C[C@H](CCC(C)=C(C)C)[C	C[C@H](CCC(=C(C)C)C)[C@H]1CC[C@@
C29H50O	CC[C@H](CC[C@@H](C)[	CC[C@H](CC[C@@H](C)[C@H]1C/C=C/[
C21H24O9	OC[C@H]1O[C@@H](OC	C1=CC(=CC=C1CCO[C@H]2[C@@H]([C@
C21H24O9	OC[C@H]1O[C@H](OCCc	C1=CC(=CC=C1CCO[C@@H]2[C@@H]([
C30H50O	CC(=C)[C@@H]1CC[C@]2	CC(=C)[C@@H]1CC[C@]2([C@H]1[C@H
C20H28O10	O[C@H]1CO[C@@H](OC[	C1[C@@H]([C@@H]([C@H]([C@@H](O
C21H24O10	OC[C@H]1O[C@H](OCCc	C1=CC(=CC=C1C(=O)O[C@@H]2[C@H]([
C21H24O10	OC[C@H]1O[C@@H](OC	C1=CC(=CC=C1C(=O)O[C@@H]2[C@H]([
C30H50O2	CC(=C)[C@@H]1CC[C@]2	CC(=C)[C@@H]1CC[C@]2([C@H]1[C@H
C21H20O11	OC[C@H]1O[C@@H](Oc2	C1=CC(=C(C=C1C2=CC(=O)C3=C(C=C(C=
C21H20O11	OCC1OC(Oc2cc(O)c3C(=O	C1=CC(=C(C=C1C2=CC(=O)C3=C(C=C(C=
C30H46O3	CC(C)=C[C@@H]1O[C@]2	CC1=C[C@@H](O[C@]23[C@@H]1[C@
C30H48O3	C[C@@H]1CC[C@@]2(C	C[C@@H]1CC[C@@]2(CC[C@@]3(C(=C
C30H48O3	CC(=C)[C@@H]1CC[C@]2	CC(=C)[C@@H]1CC[C@]2([C@H]1[C@H
C30H48O3	CC(=C)[C@@H]1CC[C@]2	CC(=C)[C@@H]1CC[C@]2([C@@H]1[C@
C30H48O3	CC(=C)[C@@H]1CC[C@@	CC(=C)[C@@H]1CC[C@]2([C@H]1[C@H
C23H26O11	OC[C@H]1O[C@@H](OC	C1=CC(=C(C=C1CCO[C@H]2[C@@H]([C
C23H26O11	OC[C@H]1O[C@@H](OC	C1=CC(=C(C=C1CCO[C@H]2[C@@H]([C
C23H26O11	OC[C@H]1O[C@H](OCCc	C1=CC(=C(C=C1CCO[C@@H]2[C@@H]([
C24H28O11	COc1cc(\C=C\C(=O)O[C@	COC1=C(C=CC(=C1)/C=C/C(=O)O[C@@H
C24H28O11	COc1cc(\C=C\C(=O)O[C@	COC1=C(C=CC(=C1)/C=C/C(=O)O[C@@H
C27H48O9	CCCCC\C=C/C/C=C\CCCC	CCCCC/C=C\C/C=C\CCCCCCCC(=O)OC[C
C27H48O9	CCCCCC=CCC=CCCCCCCC	CCCCCC=CCC=CCCCCCCCC(=O)OCC(COC
C26H32O12	OC[C@H]1O[C@@H](OC	C1[C@@]([C@H]([C@@H](O1)O[C@@H
C34H30N2O5	COc1cc2CCN(C)[C@H]3C	CN1CCC2=CC(=C3C4=C2[C@@H]1CC5=
C32H44O8	CC(=O)OC(C)(C)\C=C\C(=O	CC(=O)OC(C)(C)/C=C/C(=O)[C@@](C)([C
C35H34N2O5	COc1cc2CCN(C)[C@H]3C	CN1CCC2=CC(=C3C4=C2[C@@H]1CC5=
C32H44O9	CC(=O)OC(C)(C)\C=C\C(=O	CC(=O)OC(C)(C)/C=C/C(=O)[C@@](C)([C
C32H44O9	CC(=O)OC(C)(C)\C=C\C(=O	CC(=O)OC(C)(C)/C=C/C(=O)[C@@](C)([C
C35H58O6	CC[C@H](\C=C\[C@@H](	CC[C@H](/C=C/[C@@H](C)[C@H]1CC[C
C35H60O6	CC[C@H](CC[C@@H](C)[	CC[C@H](CC[C@@H](C)[C@H]1CC[C@
C35H60O6	CC[C@H](CC[C@@H](C)[	CC[C@H](CC[C@@H](C)[C@H]1C/C=C/[
C35H60O6	CC[C@H](CC[C@@H](C)[	CC[C@H](CC[C@@H](C)[C@H]1C/C=C/[
C34H46O9	CC(=O)O[C@@H]1C[C@@	CC(=O)O[C@@H]1C[C@]2([C@@H]3CC
C35H56O8	CC(C)=C[C@H]1C[C@](C)(	CC(=C[C@H]1C[C@]([C@@H]2[C@@H]

C35H56O8	CC(C)=CC1CC(C)(O)C2C3C	CC(=CC1CC(C2C3CCC4C5(CCC(C(C5CCC
C38H42N2O6	COc1ccc2C[C@H]3N(C)C	CN1CCC2=CC(=C3C=C2[C@H]1CC4=CC=
C29H36O15	C[C@@H]1O[C@@H](O[C	C[C@H]1[C@@H]([C@H]([C@H]([C@@
C36H58O9	CC(C)=C[C@@H]1CO[C@	CC(=C[C@@H]1CO[C@]23C[C@]4(CO2)
C31H40O15	COc1ccc(CCO[C@@H]2O	C[C@H]1[C@@H]([C@H]([C@H]([C@@
C32H42O15	COc1ccc(CCO[C@@H]2O	C[C@H]1[C@@H](O[C@H]([C@@H]([C
C32H42O15	COc1ccc(CCOC2OC(CO)C(	CC1C(OC(C(C1O)O)OC2C(C(OC(C2OC(=O
C40H64O12	CC(C)=C[C@H]1C[C@](C)(	CC(=C[C@H]1C[C@]([C@@H]2[C@H]3C
C40H64O12	CC(C)=C[C@@H]1CO[C@	CC(=C[C@@H]1CO[C@]23C[C@]4(CO2)
C40H64O12	CC(C)=C[C@H]1C[C@](C)(	CC(=C[C@H]1C[C@]([C@@H]2[C@H]3C
C40H64O12	CC(C)=C[C@@H]1CO[C@	CC(=C[C@@H]1CO[C@]23C[C@]4(CO2)
C40H64O12	CC(C)=C[C@H]1C[C@](C)(	CC(=C[C@H]1C[C@]([C@@H]2[C@@H]
C40H64O12	CC(C)=C[C@H]1C[C@](C)(	CC(=C[C@H]1C[C@]([C@@H]2[C@H]3C
C40H64O12	CC(C)=CC1CO[C@]23C[C	CC(=CC1CO[C@]23C[C@]4(CO2)[C@@H
C40H64O12	CC(C)=C[C@H]1C[C@](C)(	CC(=C[C@H]1C[C@]([C@@H]2[C@H]3C
C40H64O12	CC(C)=C[C@H]1C[C@](C)(	CC(=C[C@H]1C[C@]([C@@H]2[C@H]3C
C41H66O13	CC(C)=C[C@@H]1CO[C@	CC(=C[C@@H]1CO[C@]23C[C@]4(CO2)
C41H66O13	CC(C)=C[C@@H]1CO[C@	CC(=C[C@@H]1CO[C@]23C[C@]4(CO2)
C41H66O13	CC(C)=C[C@H]1C[C@](C)(	CC(=C[C@H]1C[C@]([C@@H]2[C@H]3C
C41H66O13	CC(C)=C[C@@H]1CO[C@	CC(=C[C@@H]1CO[C@]23C[C@]4(CO2)
C41H66O13	CC(C)=C[C@@H]1C[C@](	CC(=C[C@@H]1C[C@]([C@@H]2[C@H]
C41H66O13	CC(C)=C[C@@H]1C[C@](	CC(=C[C@@H]1C[C@]([C@@H]2[C@H]
C41H66O13	CC(C)=C[C@@H]1CO[C@	CC(=C[C@@H]1CO[C@]23C[C@]4(CO2)
C41H66O13	CC(C)=CC1CO[C@]23C[C	CC(=CC1CO[C@]23C[C@]4(CO2)[C@@H
C41H66O13	CC(C)=CC1CC(C)(O)C2C3C	CC(=CC1CC(C2C3CCC4C5(CCC(C(C5CCC
C41H68O13	CC(C)=CCC[C@](C)(O)[C@	CC(=CCC[C@@](C)([C@@H]1[C@H]2CC
C42H66O13	CC(C)=C[C@@H]1CO[C@	CC1=C2[C@H]3CC[C@@H]4[C@]5(CC[C
C42H68O14	CC(C)=C[C@@H]1CO[C@	CC(=C[C@@H]1CO[C@]23C[C@]4(CO2)
C42H68O14	CC(C)=C[C@@H]1C[C@](	CC(=C[C@@H]1C[C@]([C@H]2[C@@H]
C42H68O14	CC(C)=C[C@H]1C[C@](C)(	CC(=C[C@H]1C[C@]([C@@H]2[C@H]3C
C42H68O14	CC(C)=C[C@@H]1CO[C@	CC(=C[C@@H]1CO[C@]23C[C@]4(CO2)
C41H66O16S	CC(C)=C[C@@H]1CO[C@	CC(=C[C@@H]1CO[C@]23C[C@]4(CO2)
C41H66O16S	CC(C)=C[C@@H]1CO[C@	CC(=C[C@@H]1CO[C@]23C[C@]4(CO2)
C41H66O16S	CC(C)=C[C@@H]1CO[C@	CC(=C[C@@H]1CO[C@]23C[C@]4(CO2)
C46H74O17	CC(C)=C[C@H]1C[C@](C)(	CC(=C[C@H]1C[C@]([C@@H]2[C@H]3C
C46H74O17	CC(C)=C[C@@H]1C[C@](	CC(=C[C@@H]1C[C@]([C@H]2[C@@H]
C46H74O17	CC(C)=C[C@@H]1CO[C@	CC(=C[C@@H]1CO[C@]23C[C@]4(CO2)
C46H74O17	CC(C)=C[C@@H]1CO[C@	CC(=C[C@@H]1CO[C@]23C[C@@]4(CO
C46H74O17	CC(C)=C[C@@H]1CO[C@	CC(=C[C@@H]1CO[C@]23C[C@]4(CO2)
C46H74O17	CC(C)=C[C@@H]1CO[C@	CC(=C[C@@H]1CO[C@]23C[C@]4(CO2)
C46H74O17	CC(C)=C[C@@H]1CO[C@	CC(=C[C@@H]1CO[C@]23C[C@]4(CO2)
C46H74O17	CC(C)=C[C@@H]1CO[C@	CC(=C[C@@H]1CO[C@]23C[C@]4(CO2)
C46H74O17	CC(C)=C[C@@H]1CO[C@	CC(=C[C@@H]1CO[C@]23C[C@]4(CO2)
C46H74O17	CC(C)=CC1C[C@@](C)(O)[	CC(=CC1C[C@@]([C@@H]2[C@H]3CC[C
C46H74O17	CC(C)=CC1C[C@](C)(O)[C@	CC(=CC1C[C@]([C@@H]2[C@@H]3CC[C
C46H74O17	CC(C)=CC1COC23CC4(CO	CC(=CC1COC23CC4(CO2)C(C3C1(C)O)CC
C47H76O18	CC(C)=C[C@@H]1C[C@@	CC(=C[C@@H]1C[C@@]([C@H]2[C@@
C47H76O18	CC(C)=C[C@H]1C[C@](C)(	CC(=C[C@H]1C[C@]([C@@H]2[C@H]3C
C47H76O18	CC(C)=C[C@@H]1CO[C@	CC(=C[C@@H]1CO[C@]23C[C@]4(CO2)
C47H76O18	CC(C)=C[C@@H]1CO[C@	CC(=C[C@@H]1CO[C@]23C[C@]4(CO2)
C47H76O18	CC(C)=C[C@@H]1C[C@@	CC(=C[C@@H]1C[C@@]([C@@H]2[C@
C47H76O18	CC(C)=C[C@H]1C[C@](C)(	CC(=C[C@H]1C[C@]([C@@H]2[C@H]3C

C47H76O18	CC(C)=CC1CC(C)(O)C2C3C	CC(=CC1CC(C2C3CCC4C5(CCC(C(C5CCC
C46H74O20S	CC(C)=C[C@@H]1CO[C@	CC(=C[C@@H]1CO[C@]23C[C@]4(CO2)
C46H74O20S	CC(C)=C[C@@H]1CO[C@	CC(=C[C@@H]1CO[C@]23C[C@@]4(CO
C46H74O20S	CC(C)=C[C@@H]1CO[C@	CC(=C[C@@H]1CO[C@]23C[C@]4(CO2)
C46H74O20S	CC(C)=C[C@@H]1CO[C@	CC(=C[C@@H]1CO[C@]23C[C@]4(CO2)
C46H74O20S	CC(C)=C[C@@H]1CO[C@	CC(=C[C@@H]1CO[C@]23C[C@]4(CO2)
C47H76O21S	CC(C)=C[C@@H]1CO[C@	CC(=C[C@@H]1CO[C@]23C[C@@]4(CO
C51H82O21	CC(C)=C[C@H]1C[C@](C)(	CC(=C[C@H]1C[C@]([C@@H]2[C@H]3C
C51H82O21	CC(C)=C[C@H]1C[C@](C)(	CC(=C[C@H]1C[C@]([C@H]2[C@H]3CC[
C51H82O21	CC(C)=C[C@@H]1C[C@](	CC(=C[C@@H]1C[C@]([C@@H]2[C@]3(
C51H82O21	CC(C)=CC1C[C@](C)(O[C@	CC(=CC1C[C@]([C@@H]2[C@H]3CC[C@
C52H84O22	CC(C)=C[C@H]1C[C@](C)(	CC(=C[C@H]1C[C@]([C@@H]2[C@H]3C
C52H84O22	CC(C)=C[C@H]1C[C@](C)(	CC(=C[C@H]1C[C@]([C@@H]2[C@H]3C
C52H84O22	CC(C)=C[C@@H]1C[C@](	CC(=C[C@@H]1C[C@]([C@@H]2[C@]3(
C52H84O22	CC(C)=CC1C[C@](C)(O[C@	CC(=CC1C[C@]([C@@H]2[C@H]3CC[C@
C52H84O22	CC(C)=C[C@@H]1CO[C@	CC(=C[C@@H]1CO[C@]23C[C@]4(CO2)
C52H84O22	CC(C)=CC1C[C@@](C)(O[	CC(=CC1C[C@@]([C@@H]2[C@H]3CC[
C54H80O24	C[C@@H]1O[C@@H](O[	C[C@H]1[C@@H]([C@H]([C@H]([C@@
C54H80O24	C[C@H]1O[C@@H](O[C@	C[C@@H]1[C@H]([C@@H]([C@H]([C@
C54H80O25	C[C@@H]1O[C@@H](O[	C[C@H]1[C@@H]([C@H]([C@H]([C@@
C54H80O25	C[C@H]1O[C@@H](O[C@	C[C@@H]1[C@H]([C@@H]([C@H]([C@

from Datawarrior tool

Molweight	cLogP	cLogS	H-Acceptor	H-Donors	Druglikene:	Mutagenic	Tumorigeni
124.139	0.7166	-1.204	2	2	-2.2456	high	none
138.122	-2.855	-1.333	3	1	-5.5215	none	none
196.245	0.9068	-1.92	3	1	-4.2161	none	none
208.212	1.3339	-2.035	4	1	0.27506	none	none
208.212	1.3339	-2.035	4	1	0.27506	none	none
270.239	2.3357	-2.856	5	3	0.28194	high	none
286.238	1.99	-2.56	6	4	0.28194	none	none
290.354	0.6592	-1.844	6	4	-18.542	none	high
290.354	0.6592	-1.844	6	4	-18.542	none	high
302.237	1.4902	-2.491	7	5	-0.08283	high	high
316.305	-1.0361	-0.72	8	6	-4.6724	none	none
316.305	-1.0361	-0.72	8	6	-4.6724	none	none
356.326	-0.7789	-1.421	9	5	-5.8493	none	none
370.417	-0.9556	-0.698	9	4	-13.857	none	high
370.417	-0.9556	-0.698	9	4	-13.857	none	high
398.672	7.9248	-6.099	1	1	-5.0079	none	none
414.715	8.6758	-6.558	1	1	-5.6667	none	none
420.413	0.8923	-2.3	9	5	-1.455	none	none
420.413	0.8923	-2.3	9	5	-1.455	none	none
426.726	7.6469	-6.803	1	1	-22.172	none	none
428.432	-1.2487	-1.546	10	6	-4.6719	none	none
436.412	0.5466	-2.004	10	6	-1.455	none	none
436.412	0.5466	-2.004	10	6	-1.455	none	none
442.725	6.7202	-6.296	2	2	-23.933	none	none
448.379	7.00E-04	-2.446	11	7	-3.2535	none	none
448.379	7.00E-04	-2.446	11	7	-3.2535	none	none
454.692	6.1234	-5.829	3	1	-0.45158	none	none
456.708	6.0021	-6.111	3	2	-3.658	none	none
456.708	6.3706	-6.278	3	2	-21.111	none	none
456.708	6.3706	-6.278	3	2	-21.111	none	none
456.708	6.3706	-6.278	3	2	-21.49	none	none
478.448	0.5301	-2.078	11	7	-3.1704	none	none
478.448	0.5301	-2.078	11	7	-2.0433	none	none
478.448	0.5301	-2.078	11	7	-3.1704	none	none
492.475	0.8058	-2.392	11	6	-2.9905	none	none
492.475	0.8058	-2.392	11	6	-2.9905	none	none
516.669	3.9699	-3.815	9	5	-28.768	none	high
516.669	3.9699	-3.815	9	5	-28.768	none	high
536.528	-0.1157	-2.156	12	6	-0.54316	none	none
546.621	6.8386	-9.35	7	1	4.9691	low	none
556.693	3.3832	-4.694	8	3	-4.6016	none	none
562.664	7.0099	-8.195	7	1	4.6261	low	none
572.692	2.5311	-4.295	9	4	-4.6795	none	none
572.692	2.5311	-4.295	9	4	-4.6795	none	none
574.84	5.7659	-6.141	6	4	-2.8093	none	none
576.855	6.0181	-6.369	6	4	-8.3009	none	none
576.855	6.8387	-6.258	6	4	-9.4747	none	none
576.855	6.8387	-6.258	6	4	-9.4747	none	none
598.73	3.8678	-5.104	9	2	-4.5012	none	none
604.822	4.1694	-5.472	8	4	-4.7063	none	none

604.822	4.1694	-5.472	8	4	-4.7063	none	none
622.759	6.501	-7.334	8	0	4.6496	none	none
624.59	-0.3803	-2.285	15	9	-0.5767	none	none
634.848	3.512	-5.125	9	5	-4.7595	none	none
652.644	0.1711	-2.913	15	7	-0.415	none	none
666.67	0.3658	-2.965	15	7	-3.7289	none	none
666.67	0.3658	-2.965	15	7	-3.7289	none	none
736.936	2.8424	-5.301	12	6	-5.6748	none	none
736.936	2.787	-5.083	12	6	-2.5874	none	none
736.936	2.9343	-5.301	12	6	-5.4857	none	none
736.936	2.787	-5.083	12	6	-6.0274	none	none
736.936	2.8424	-5.301	12	6	-5.6748	none	none
736.936	2.9343	-5.301	12	6	-5.4857	none	none
736.936	2.787	-5.083	12	6	-6.0274	none	none
736.936	2.9343	-5.301	12	6	-5.4857	none	none
736.936	2.8424	-5.301	12	6	-2.2348	none	none
766.962	2.2769	-4.954	13	7	-4.4957	none	none
766.962	2.2769	-4.954	13	7	-4.4957	none	none
766.962	2.3323	-5.172	13	7	-4.151	none	none
766.962	2.185	-4.954	13	7	-4.0453	none	none
766.962	2.2404	-5.172	13	7	-3.689	none	none
766.962	2.3323	-5.172	13	7	-4.151	none	none
766.962	2.185	-4.954	13	7	-4.0616	none	none
766.962	2.185	-4.954	13	7	-4.0453	none	none
766.962	2.2404	-5.172	13	7	-3.689	none	none
768.978	2.7884	-4.977	13	8	-17.532	none	none
778.973	2.5647	-4.975	13	7	-5.4262	none	none
796.988	1.6749	-4.825	14	8	-4.7595	none	none
796.988	1.7303	-5.043	14	8	-4.4097	none	none
796.988	1.7303	-5.043	14	8	-4.4097	none	none
796.988	1.6749	-4.825	14	8	-4.7595	none	none
847.025	0.6621	-3.808	16	7	0.045906	none	none
847.025	0.6621	-3.808	16	7	0.045906	none	none
847.025	0.5702	-3.808	16	7	-2.4025	none	none
899.077	1.0053	-5.001	17	9	-6.3015	none	none
899.077	1.0053	-5.001	17	9	-6.3015	none	none
899.077	0.9499	-4.783	17	9	-6.6463	none	none
899.077	0.9499	-4.783	17	9	-6.6463	none	none
899.077	0.9499	-4.783	17	9	-6.6463	none	none
899.077	0.9499	-4.783	17	9	-6.6463	none	none
899.077	0.8926	-4.783	17	9	-7.3504	none	none
899.077	0.9499	-4.783	17	9	-6.6463	none	none
899.077	0.9499	-4.783	17	9	-6.6463	none	none
899.077	1.0053	-5.001	17	9	-6.3015	none	none
899.077	1.0053	-5.001	17	9	-6.3015	none	none
899.077	0.9499	-4.783	17	9	-6.6463	none	none
929.102	0.4033	-4.872	18	10	-6.5608	none	none
929.102	0.4033	-4.872	18	10	-6.5608	none	none
929.102	0.3479	-4.654	18	10	-6.9107	none	none
929.102	0.3479	-4.654	18	10	-6.9107	none	none
929.102	0.4033	-4.872	18	10	-6.5608	none	none
929.102	0.4033	-4.872	18	10	-6.5608	none	none

929.102	0.4033	-4.872	18	10	-6.5608	none	none
979.14	-0.6649	-3.637	20	9	-2.0776	none	none
979.14	-0.6649	-3.637	20	9	-2.0776	none	none
979.14	-0.6649	-3.637	20	9	-2.0776	none	none
979.14	-0.6649	-3.637	20	9	-2.0776	none	none
979.14	-0.6649	-3.637	20	9	-2.0776	none	none
1009.17	-1.2669	-3.508	21	10	-2.3326	none	none
1031.19	-0.2298	-4.83	21	11	-11.832	none	none
1031.19	-0.2298	-4.83	21	11	-11.832	none	none
1031.19	-0.2298	-4.83	21	11	-11.832	none	none
1031.19	-0.2298	-4.83	21	11	-11.832	none	none
1061.22	-0.8318	-4.701	22	12	-11.832	none	none
1061.22	-0.8318	-4.701	22	12	-11.832	none	none
1061.22	-0.8318	-4.701	22	12	-11.832	none	none
1061.22	-0.8318	-4.701	22	12	-11.832	none	none
1061.22	-0.8872	-4.483	22	12	-11.977	none	none
1061.22	-0.8318	-4.701	22	12	-11.832	none	none
1113.21	-0.9997	-4.766	24	11	-3.3349	none	none
1113.21	-0.9997	-4.766	24	11	-3.3349	none	none
1129.2	-1.8518	-4.367	25	12	-3.445	none	none
1129.2	-1.8518	-4.367	25	12	-3.445	none	none

Reproducti	Irritant	DrugScore
none	high	0.192684
none	none	0.492461
none	none	0.485792
none	none	0.744349
none	none	0.744349
none	none	0.418889
none	none	0.709568
none	low	0.224854
none	low	0.224854
none	none	0.241775
none	none	0.475719
none	none	0.475719
none	none	0.456496
none	low	0.217332
none	low	0.217332
none	none	0.146393
none	none	0.130941
none	none	0.49187
none	none	0.49187
none	none	0.129817
none	high	0.253534
none	none	0.485366
none	none	0.485366
none	none	0.145647
none	none	0.411052
none	none	0.411052
none	high	0.138089
none	none	0.164992
none	none	0.148929
none	none	0.148929
none	none	0.148929
none	none	0.394199
none	none	0.422406
none	none	0.394199
none	none	0.382309
none	none	0.382309
none	none	0.166795
none	none	0.166795
low	none	0.369163
none	none	0.156514
none	low	0.19505
none	none	0.152708
none	low	0.20961
none	low	0.20961
none	none	0.139368
none	none	0.122218
none	none	0.11151
none	none	0.11151
none	low	0.161504
none	high	0.10851

none	high	0.10851
none	none	0.189929
none	none	0.388804
none	high	0.117551
none	none	0.374227
none	none	0.268516
none	none	0.268516
none	high	0.107304
none	high	0.119065
none	high	0.106878
none	high	0.111548
none	high	0.107304
none	high	0.106878
none	high	0.111548
none	high	0.106878
none	high	0.117276
none	high	0.115418
none	high	0.115418
none	high	0.111567
none	high	0.116428
none	high	0.112855
none	high	0.111567
none	high	0.116396
none	high	0.116428
none	high	0.112855
none	high	0.111451
none	low	0.150127
none	high	0.11789
none	high	0.114023
none	high	0.114023
none	high	0.11789
none	high	0.202073
none	high	0.202073
none	high	0.144869
none	high	0.11259
none	high	0.11259
none	high	0.11666
none	high	0.11666
none	high	0.11666
none	high	0.11666
none	high	0.116639
none	high	0.11666
none	high	0.11666
none	high	0.11259
none	high	0.11259
none	high	0.11666
none	high	0.115145
none	high	0.115145
none	high	0.119163
none	high	0.119163
none	high	0.115145
none	high	0.115145

none	high	0.115145
none	high	0.149928
none	high	0.149928
none	high	0.149928
none	high	0.149928
none	high	0.149928
none	high	0.148467
none	high	0.115569
none	high	0.115569
none	high	0.115569
none	high	0.115569
none	high	0.118033
none	high	0.118033
none	high	0.118033
none	high	0.118033
none	high	0.12196
none	high	0.118033
none	low	0.161086
none	low	0.161086
none	low	0.170428
none	low	0.170428
