# Supplementary material for: Bio-Computational Evaluation of Compounds of Bacopa Monnieri as a Potential Treatment for Schizophrenia
Source: Molecules. 2022 Oct 19;27(20):7050. doi: 10.3390/molecules27207050 (PMC9611144; doi:10.3390/molecules27207050)
Supplement: Supplementary file 1 [file molecules-27-07050-s001.zip › Table S3.pdf]

Table S3: Selected natural compounds chemical and structural information.

S.No.	Natural compounds IDs	IUPAC name	SMILES	2D Structures
1.	Drug as a Control Quetiapine	2-[2-(4-benzo[b][1,4]benzothiazepin-6-ylpiperazin-1-yl)ethoxy]ethanol	C1CN(CCN1CCOCCO)C2=NC3=CC=CC=C3SC4=CC=CC=C42	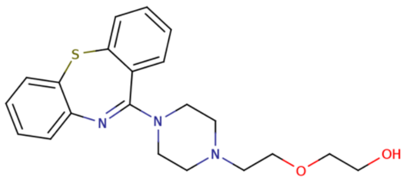
2.	STXBP1_CID:5319292 Martynoside	[(2*R*,3*R*,4*R*,5*R*,6*R*)-5-hydroxy-6-[2-(3-hydroxy-4-methoxyphenyl)ethoxy]-2-(hydroxymethyl)-4-[(2*S*,3*R*,4*R*,5*R*,6*S*)-3,4,5-trihydroxy-6-methyloxan-2-yl]oxyoxan-3-yl] (*E*)-3-(4-hydroxy-3-methoxyphenyl)prop-2-enoate	CC1C(C(C(C(O1)OC2C(C(OC(C2OC(=O)C=CC3=CC(=C(C=C3)O)OC)CO)OCCC4=CC(=C(C=C4)OC)O)O)O)O)O	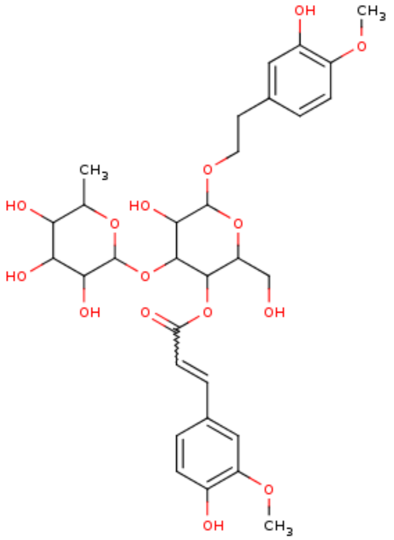

3.	STXBP1_CID:5281800<br>Acteoside	[(2*R*,3*R*,4*R*,5*R*,6*R*)-6-[2-(3,4-dihydroxyphenyl)ethoxy]-5-hydroxy-2-(hydroxymethyl)-4-[(2*S*,3*R*,4*R*,5*R*,6*S*)-3,4,5-trihydroxy-6-methyloxan-2-yl]oxyoxan-3-yl] (*E*)-3-(3,4-dihydroxyphenyl)prop-2-enoate	CC1C(C(C(C(O1)OC2C(C(OC(C2OC(=O)C=CC3=CC(=C(C=C3)O)O)CO)OCCC4=CC(=C(C=C4)O)O)O)O)O)O	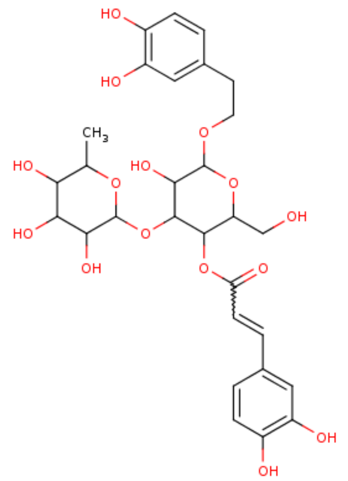
4.	STXBP1_CID:44559250<br>Dehydroapateline	(21*S*)-27-methoxy-22-methyl-15,29,31-trioxa-7,22-diazaoctacyclo[19.9.3.$2^{16,19}$.$1^{4,30}$.$1^{10,14}$.$0^{3,8}$.$0^{25,33}$.$0^{28,32}$]heptatriaconta-1(30),2,4(34),7,10(37),11,13,16,18,25,27,32,35-tridecaen-13-ol	CN1CCC2=CC(=C3C4=C2C1CC5=CC=C(C=C5)OC6=C(C=CC(=C6)CC7=NCCC8=CC(=C(O4)C=C87)O3)O)OC	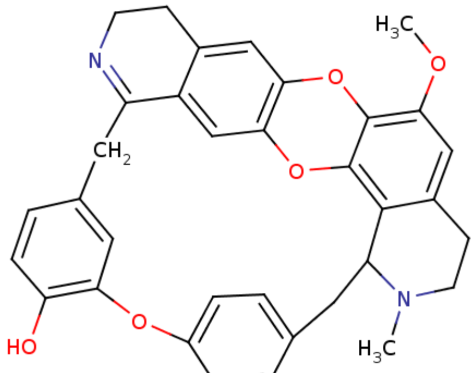

5.	STXBP1_CID:5291488 Luteolin 7-galactoside	2-(3,4-dihydroxyphenyl)-5-hydroxy-7-[3,4,5-trihydroxy-6-(hydroxymethyl)oxan-2-yl]oxychromen-4-one	C1=CC(=C(C=C1C2=CC(=O)C3=C(C=C(C=C3O2)OC4C(C(C(C(O4)CO)O)O)O)O)O)O	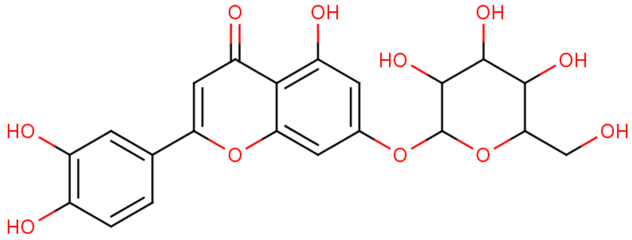
6.	STXBP1_CID:11145924 Bacopaside C	[(3*S*,4*R*,5*S*)-5-[(2*R*,3*R*,4*S*,5*S*,6*R*)-4,5-dihydroxy-6-(hydroxymethyl)-2-(2-phenylethoxy)oxan-3-yl]oxy-3,4-dihydroxyoxolan-3-yl]methyl 4-hydroxybenzoate	C1C(C(C(O1)OC2C(C(C(OC2OCCC3=CC=CC=C3)CO)O)O)O)(COC(=O)C4=CC=C(C=C4)O)O	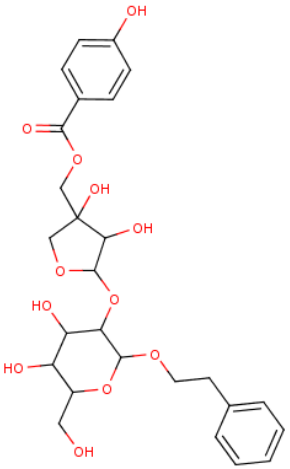
7.	STXBP1_CID:15922618 Bacopaside III	[(2*R*,3*S*,4*S*,5*R*,6*S*)-6-[(2*S*,3*R*,4*S*,5*S*)-3,5-dihydroxy-2-[[(1*S*,2*R*,5*R*,7*S*,10*R*,11*R*,14*R*,15*S*,16*S*,17*R*,20*R*)-16-hydroxy-2,6,6,10,16-pentamethyl-17-(2-methylprop-1-enyl)-19,21-dioxahexacyclo[18.2.1.$0^{1,14}$.$0^{2,11}$.$0^{5,10}$.$0^{15,20}$]tricosan-7-yl]oxy]oxan-4-yl]oxy-3,4,5-trihydroxyoxan-2-yl]methyl hydrogen sulfate	CC(=CC1COC23CC4(CO2)C(C3C1(C)O)CCC5C4(CCC6C5(CCC(C6(C)C)OC7C(C(C(CO7)O)OC8C(C(C(C(O8)COS(=O)(=O)O)O)O)O)O)C)C)C	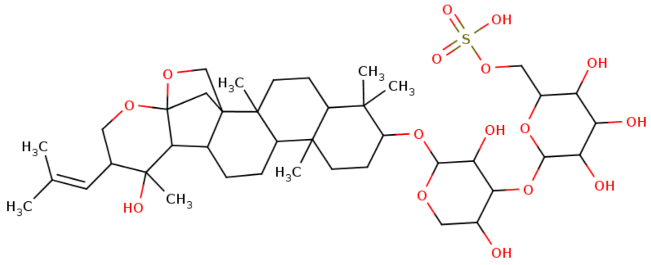

8.	STXBP1_CID:11091080<br>Monnieraside I	[(2*R*,3*R*,4*S*,5*S*,6*R*)-4,5-dihydroxy-6-(hydroxymethyl)-2-[2-(4-hydroxyphenyl)ethoxy]oxan-3-yl] 4-hydroxybenzoate	C1=CC(=CC=C1CCOC2C(C(C(C(O2)CO)O)O)OC(=O)C3=CC=C(C=C3)O)O	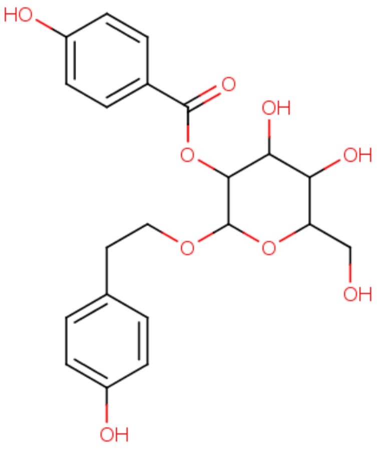
9.	STXBP1_CID:9847922<br>Plantainoside B	[(2*R*,3*R*,4*S*,5*S*,6*R*)-2-[2-(3,4-dihydroxyphenyl)ethoxy]-4,5-dihydroxy-6-(hydroxymethyl)oxan-3-yl] (*E*)-3-(3,4-dihydroxyphenyl)prop-2-enoate	C1=CC(=C(C=C1CCOC2C(C(C(C(O2)CO)O)O)OC(=O)C=CC3=CC(=C(C=C3)O)O)O)O	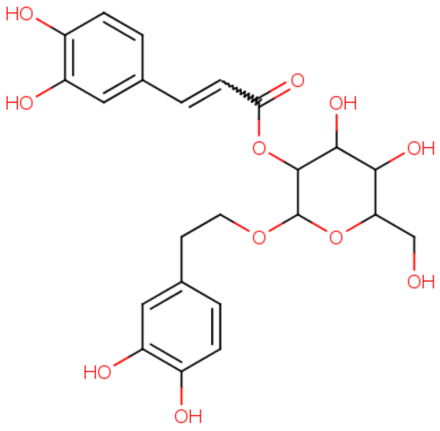
10.	STXBP1_CID:163188454<br>NA	[(2*R*,3*R*,4*R*,5*R*,6*R*)-4-[(2*S*,3*R*,4*R*,5*R*,6*R*)-3,4-dihydroxy-6-(hydroxymethyl)-5-methyloxan-2-yl]oxy-5-hydroxy-6-[2-(3-hydroxy-4-methoxyphenyl)ethoxy]-2-(hydroxymethyl)oxan-3-yl] (*E*)-3-(4-hydroxy-3-methoxyphenyl)prop-2-enoate	CC1C(OC(C(C1O)O)OC2C(C(OC(C2OC(=O)C=CC3=CC(=C(C=C3)O)OC)CO)OCCC4=CC(=C(C=C4)OC)O)O)CO	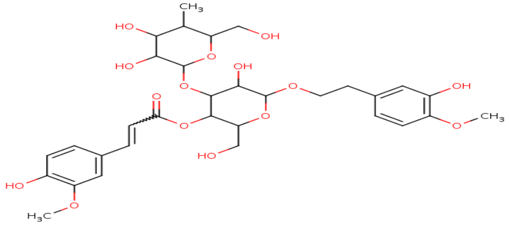
